# Pathways to Breast Cancer Diagnosis and Treatment Among Women in Ghana: A Qualitative Study

**DOI:** 10.1089/whr.2020.0117

**Published:** 2021-07-16

**Authors:** Waruiru Mburu, Adwoa Bemah Boamah Mensah, Beth Virnig, John H. Amuasi, Baffour Awuah, Carolyn M. Porta, Ernest Osei-Bonsu, Shalini Kulasingam

**Affiliations:** ^1^ Division of Epidemiology and Community Health, University of Minnesota, Minneapolis, Minnesota, USA.; ^2^ Department of Nursing, Kwame Nkrumah University of Science and Technology, Kumasi, Ghana.; ^3^ Division of Health Policy and Management, University of Minnesota, Minneapolis, Minnesota, USA.; ^4^ Department of Global Health, School of Public Health, Kwame Nkrumah University of Science and Technology, Kumasi, Ghana.; ^5^ Ministry of Health, Accra, Ghana.; ^6^ Department of Medical Oncology and Radiation, Komfo Anokye Teaching Hospital, Kumasi, Ghana.; ^7^ School of Nursing, University of Minnesota, Minneapolis, Minnesota, USA.

**Keywords:** breast cancer, early detection, pathways of care, Ghana, sub-Saharan Africa

## Abstract

***Background:*** Breast cancer is the leading cause of cancer death among Ghanaian women and most women are identified once they develop symptoms. Women then must navigate a complex health care system to get diagnosed and receive orthodox medicine. We describe Ghanaian women's pathways of care from breast cancer-related symptom detection to treatment receipt.

***Methods:*** We conducted a qualitative study using an empirical phenomenological approach. We used a purposive sampling technique to recruit 31 women with breast cancer who were receiving treatment at Komfo Anokye Teaching Hospital in Kumasi, Ghana. They participated in semistructured in-depth interviews between November 2019 and March 2020. All interviews were transcribed verbatim and analyzed using a deductive coding approach.

***Results:*** Women navigate approximately nine steps from symptom detection to receiving orthodox breast cancer treatment. The breast cancer care pathway is not linear and women frequently move among different management approaches, including alternative therapy (faith healing and traditional herbal healing). All the women detected the symptoms themselves. Some of the women sought orthodox medicine due to information from the media.

***Conclusions:*** Alternative therapy providers play a critical role in the breast cancer diagnosis and care pathways in Ghana underscoring the need to formally integrate them into the health care system. Breast cancer awareness programs through the media and educational programs aimed at alternative therapy providers may reduce the time from symptom detection to receipt of orthodox medicine.

## Introduction

Breast cancer is the leading cause of cancer death among women worldwide. In sub-Saharan Africa, breast cancer represents 25% of the total cancer burden making it the most common cancer and leading cause of cancer deaths among women.^1^ Breast cancer is the most common cancer among women in Ghana with an age-standardized incidence rate of 43.0 per 100,000.^2^

Most breast cancer patients in Ghana are diagnosed with advanced metastatic disease due to delays in formal diagnosis. Several studies report that time from symptom onset to diagnosis and treatment receipt varies substantially in Ghana. In one study, time between detecting a symptom and reporting to a hospital ranged from 1 week to 5 years,^3^ whereas another study reported an average of 10.8 months between symptom detection and diagnosis.^4^ Dedey et al. found that the median time between a breast cancer diagnosis and start of definitive treatment was 5 weeks.^5^ Factors associated with these diagnostic and treatment delays included misdiagnosis in previous medical consultation, financial constraints, and lack of adequate information from the health care workers.^3,5^

Ghana, like most sub-Saharan African countries, lacks a national breast cancer screening program. Thus, most women seek breast cancer care once they identify symptoms.^3^ The women then have to navigate the health care system to get diagnosed and receive treatment. To our knowledge, only one study, based in Malawi, has detailed women's specific pathways to breast cancer diagnosis and treatment in sub-Saharan Africa.^6^ Three studies set in Ghana have described some aspects of the breast cancer care-seeking pathway in Ghana. Two studies described women's symptom recognition, appraisal and intent to seek medical care, whereas the third described the diagnosis and treatment procedures.^4,7,8^ However, no study has outlined in detail the pathways that women in Ghana navigate from symptom detection through treatment receipt. Understanding this full process and appreciating women's understanding and needs is essential if effective interventions are to be developed.

The objective of this study is to explore Ghanaian women's pathways from breast cancer-related symptom detection to treatment. Our definition of the term ‘pathways’ is guided by the Model of Pathways to Treatment Framework.^9^ We define pathways as the sequence of events and processes in a woman's journey from breast cancer-related symptom(s) detection to treatment receipt. According to the Model of Pathways to Treatment Framework, events are the “key time periods,” whereas processes are the “cognitive, emotional, behavioral, organizational, or structural actions” in the woman's journey to treatment receipt.^9^ Based on the findings from the interviews, we present a framework showing specific steps in the pathways and how women transition from one step to another.

## Materials and Methods

### Theoretical framework

The Model of Pathways to Treatment framework informed our study development and analysis.^9^ The model has four intervals: appraisal, help seeking, diagnostic, and pretreatment. The appraisal interval is the time between discovering a symptom and perceiving a reason to seek help from a health care provider. The help-seeking interval is the time period between perceiving the reason to seek help from a health care provider to visiting a health care provider. In our study, we combined the appraisal and help-seeking intervals to form the detection interval. This was based on similar breast cancer studies, which have found it challenging to distinguish appraisal and help-seeking intervals.^10^ The diagnostic interval is the time between seeing a health care provider and being diagnosed with cancer. The pretreatment interval is the period between getting diagnosed and starting treatment. We also added the treatment interval to have a comprehensive understanding of the care continuum.

### Study design and setting

An empirical phenomenological approach was used to explore the pathways of women diagnosed with breast cancer from the moment they detected symptoms to treatment. Phenomenology is used to describe and understand the lived experiences of a group of individuals grounded in the individuals' description and meaning.^11^ The aim of our study was to describe the perspectives and lived experiences of our research participants from breast cancer-related symptom detection to treatment receipt. Given our interest in describing the phenomenon of navigating breast cancer diagnosis and treatment processes and from the perspective of our study participants, a phenomenological design and methodological approach was chosen for our study. This study was conducted in Kumasi, Ghana. Participants were recruited from the Komfo Anokye Teaching Hospital (KATH) Oncology Department. KATH is the second largest hospital in Ghana and the main cancer management hospital in the Ashanti region of Ghana.

### Study population, sampling, and sample size

Eligible participants were women (18 years and older) with a histopathology-confirmed diagnosis of breast cancer, who had started receiving at least one breast cancer treatment (surgery, chemotherapy, or radiotherapy), could speak Twi (local language), and were in a stable condition at the time of study. We reviewed the patients' medical records to confirm diagnosis and treatment receipt. We used a purposive sampling technique^12^ to enroll participants who had experienced the phenomenon under study, were willing to participate and could clearly communicate their breast cancer management experiences. Data saturation was determined from the interviews. We determined we had reached data saturation when data from additional interviews tended to be redundant of the data we had already collected.^13,14^ By the 27th interview there was no new information. We carried out four more interviews to confirm that we had reached data saturation for a total of 31 interviews.

### Recruitment

A trained Research Assistant (RA) met the patients at the KATH Oncology Department to assess eligibility and invite them to participate in the study. Specifically, the RA read to each potential participant the study information sheet, which included the goals of the study, what is required of each participant, and a reminder that participation was voluntary. The interviews were carried out between November 2019 and March 2020.

### Data collection

A semistructured interview guide was used for the interview. The guiding questions were based on previous similar publications^6,10,15–20^ and were adapted for use in the Ghana context. Section 1 of the interview guide (Supplementary Data) was semistructured, and it was based on the Model of Pathways to Treatment framework. Section 2 (Supplementary Data) included patient demographics and was close ended. All participants gave consent by signing or thumb printing. Interviews were carried out in Twi in a private room within the KATH Oncology Department. We pretested the interview guide by interviewing three breast cancer patients from the Oncology Department and a Ghanaian qualitative researcher with clinical experience in oncology (A.B.B.M.) reviewed the guiding questions for sociocultural appropriateness. Based on the pretest interviews and advice from A.B.B.M., we revised the order of the interview guide questions. The main guiding question was: Please tell us the story of your journey from when you detected bodily change to when you started receiving breast cancer treatment. We then used more specific follow-up questions to further understand the events in each interval. Interviews lasted 42 minutes on average (range 19–84 minutes).

### Data analysis

Data collection and analysis were undertaken concurrently. All interviews were audiorecorded to ensure we captured all the patient's information and to enable transcription for subsequent analysis by independent analysts. At the end of each interview, the interviewer and one of the authors (W.M.) reviewed the recording and saved it to a password-protected laptop. All interviews were conducted in Twi and translated into English by a professional transcriber who is proficient in Twi and English. One of the authors who is proficient in Twi and English (A.B.B.M.) reviewed a random sample of the recordings and transcripts to ensure accuracy in interviewing, transcription, and translation. We used NVivo 12plus^21^ computer software to facilitate analysis organization.

A deductive coding analytic process was used.^22^ We had a list of codes (“start list”) based on the Model of Pathways to Treatment,^9^ but we also created codes for other concepts that emerged from the data and were not part of the start list. Two analysts (W.M. and A.B.B.M.) independently coded the transcripts in three stages. For the first stage, they independently assigned codes to text sections based on the Model of Pathways to Treatment framework.^9^ For the second stage, they independently re-reviewed the data to identify emerging codes that were not part of the framework. They then discussed the codes, clarified discrepancies, revised definitions. and created new codes. For the third stage, the analysts jointly organized the codes into steps and themes guided by the Model of Pathways to Treatment Framework.^9^ The rest of the authors confirmed the findings. This in-depth analysis process ensured reliability of the results.^23^

### Trustworthiness and credibility

Credibility was achieved by triangulation^24^ and confirmation that the transcripts reflected the participants' experiences.^25^ Data source triangulation involved using information from the patient's medical records at KATH to verify the procedures and treatments that the patients received. Analyst triangulation involved two of the authors (A.B.B.M. and W.M.) comparing and discussing the analyses until consensus was achieved. Immediately after each interview, the interviewer summarized the interview for each participant and asked them to confirm that it reflected their experiences. In addition, five randomly selected participants independently reviewed their transcripts to affirm that the transcripts accurately reflected the interview content. This was done within 1 week of transcription. No participant offered corrections or expressed concerns about the content of the interviews. Field notes, which included the participants' nonverbal cues, concerns, and interviewers' reflections, were recorded after each interview and referred to during the analysis. The two RAs who carried out the interviews are nurses by training, thus have a clinical understanding of breast cancer. However, none of them work in the KATH Oncology Department and had no direct relationship with the participants.

Ethics approval was granted by the Institutional Review Boards at KATH (KATH-IRB/AP/001/19) and University of Minnesota (STUDY00006750). We deidentified transcripts before analysis to ensure anonymity. Audio records and transcripts (without any identifying information) were stored on a password-protected computer. We followed the Standards for Reporting Qualitative Research.^26^

## Results

We interviewed 31 women who were diagnosed with breast cancer between 2015 and 2019. The mean age of the participants was 51 years. Most of the participants were married/partnered/cohabiting, Christians, had at least primary school education and had no family history of cancer. Of those who had a job (*n* = 14), most of them were self-employed. Thirty of the 31 participants had health insurance (Table 1).

**Table 1.** Sociodemographic Characteristics of the Sample (*N* = 31)

Characteristic	*n* (%)
Age in years, mean (range)	51 (28–72)
Marital status	
Married/partnered/cohabiting	22 (71)
Widowed/divorced/separated	9 (29)
Highest education level	
Primary school	18 (58)
High school	3 (10)
Technical college diploma	5 (16)
Bachelors degree	2 (6)
Other	3 (10)
Religion	
Christian	28 (90)
Muslim	3 (10)
Employed	
Yes	14 (45)
No	17 (55)
Employed Yes	14 (45)
Type of employment	
Government	3 (21)
Self-employed	10 (71)
Other	1 (7)
Have health insurance	
Yes	30 (97)
No	1 (3)
Family cancer history	
Yes	11 (35)
No	20 (65)

**FIG. 1.**
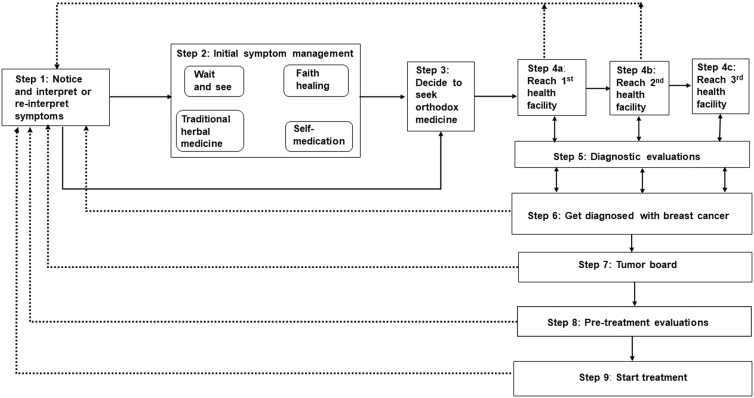
Pathway of care from symptom detection to treatment for breast cancer patients in Kumasi, Ghana. Dotted lines represent patients going back to previous steps. Solid lines represent patients moving forward in the pathway.

We describe the pathway steps that emerged from each interval and provide more sample quotes in Table 2. The pathway was not linear and some patients looped back to earlier steps, whereas some skipped some of the steps (Fig. 1).

**Table 2.** Breast Cancer Pathway Steps and Sample Quotes

Step	Substep	Data sample
Step 1: Initial symptom recognition and appraisal	Type of symptom and way of discovery	“I was there one day and I examined my breast and I realized that there is a lump at the top.” (P21)
		“When I woke up in the morning, I wore a white nightie the previous night and I realized there was blood where my breast touched the fabric.” (P19)
	Initial symptom interpretation	“I felt the lump so I kept touching it. My mind went there…. [assuming it was due to breast cancer].” (P8)
		“Whenever I am about to have my menses, my breasts will swell up and become bigger… When I observed this change… I reasoned that it could be related to my menses. I did not perceive it serious.” (P11)
	Symptom disclosure	“I showed it to three of my sisters…. It looked as if the breast was tilted…One of my sisters advised me to take it straight to [a district hospital] in the morning if I have insurance.” (P12)
		“There was a nurse in our house and…when I told her, she told me to wait for a while because it could be the result of the ‘sure’ [deodorant] I am using, so I should stop using it.” (P18)
Step 2: Initial management before seeking orthodox medicine	Traditional herbal medicine	“I considered the lump as one of the usual breast problems. So, I started treating it with herbal medications.” (P9)
	Over-the-counter medication	“My children…bought me some medication from the chemist to stop the breast pains.” (P32)
	Faith healing	“I initially thought the breast heaviness was due to evil witches bedeviling me. So I went to my personal priest…and resorted to prayers.” (P26)
	Wait and see	“I found the lump in August, I thought it was just an ordinary lump…. Hence I waited until around October but monitored the symptom alongside.” (P6)
Step 3: Decision to seek orthodox medicine	Additional symptoms	“Since it started being painful I had to do away with the herbal treatment and report to the [hospital].” (P24)
	Alternative therapy failure	“When the lump appeared, we started treating it with herbal medications and it went down a bit and it reappeared a little worse…When the herbal medication did not work we brought it to [hospital].” (P9)
	Advice and pressure from social network	“The moment I told them [friends about symptoms], they said ‘be quick and take it to the hospital because you don't play around with diseases affecting the breast….’ And I rushed to the hospital.” (P7)
Step 4a: Reach first health facility		“And when we went there [district hospital] the doctor declared it to be a lump so we should go and take a scan and he will take it (the lump) out for me.” (P17)
		“I went to see the doctor [district hospital] and he told me that those who used to work on these kind of cases are no longer working at the hospital. So he called another doctor at [tertiary hospital] and informed him that he is sending a patient to him…I started coming here [tertiary hospital].” (P30)
Step 4b: Reach second health facility		“I went to the hospital [regional facility], and they told me ‘No, it is not anything’ so I should go home…Three weeks' time, I went to [another facility] … for review. And when they checked, they said the thing [lump] was no longer there. And me too, I just relaxed and stayed at home.” (P16)
Step 5: Diagnostic evaluations		“Well, when I came to [tertiary hospital] he [doctor] gave us some tests and we took a very big picture [scans]. He also requested another test at [private lab]. I have even been to that place two times. And all the documents associated with these are there. And I can't even count the numerous other tests I've done over here.” (P5)
		“He [doctor] took a sample of the breast…then he asked that I take a scan of the breast. He also asked me to go to [private lab] where they put the breast in a machine (mammogram].” (P11)
Step 6: Get diagnosed with breast cancer		“I did the breast scan mammogram and the tissue biopsy…and I went for the results and it they told me it was cancer.” (P8)
Step 7: Discussion at tumor board		“Following the diagnosis, we were scheduled for a meeting [tumor board] in two weeks. The doctor explained that everyone involved [at the tumor board] will bring their opinion …it is important for me to sit in during deliberations… However, the same doctor later told us not to come for the meeting due to my long distance, so he will represent me.” (P4)
Step 8: Evaluations before starting treatment		“He requested I go for a lab test… they examined a lot of tissues They examine your blood; they check the blood to know if you can take the thing [chemotherapy].” (P23)
Step 9: Start treatment		“I did four chemotherapy cycles before I was operated on. Then another four after operation.” (P12)

### Detection interval

The average time between symptom detection and seeking orthodox medicine was 11 months (range 1 day–13 months).

### Step 1: Initial symptom recognition and appraisal

#### Type of symptom and way of discovery

All the women detected initial symptoms themselves. The most common initial symptom was lump (*n* = 23). Other initial symptoms included swollen breast (*n* = 3), fluid oozing from the breast nipple (*n* = 2), burning sensation (*n* = 1), rashes on the breast (*n* = 1), and pain (*n* = 1). Two of the women recognized the initial symptom while doing a self-breast exam, whereas the other 29 discovered them accidentally.

#### Initial symptom interpretation

Only two women thought the initial symptom was due to breast cancer. Both of these women had aunts who died of breast cancer and one of them was a nurse, thus they were aware of breast cancer.

“In my mind, I thought maybe I have been afflicted with breast cancer… because my mother's sister had it.” (P3)

The other 29 women did not think the initial symptom was serious. Most of them thought it was a boil while others associated the symptoms to the menstrual cycle, insect bite, food they had eaten, or breast-feeding.

“It was like Oh this is just like a lump…. I did not anticipate that it was serious or going to be breast cancer.” (P6)

#### Symptom disclosure

The women disclosed their symptoms to individuals they trusted, which included relatives, friends, who were in the health care field, and work colleagues. Most of the people advised the women to go to the hospital for a checkup, others advised them to wait and monitor the symptoms, whereas some advised them to see a traditional healer.

“And when I saw it [blood oozing from nipple], I informed a sister living in the same house. She advised going for herbal medicine.” (P19)

### Step 2: Initial symptom management before seeking orthodox care

Only two patients sought orthodox medicine right after discovering the symptoms (*i.e.*, Step 1 to Step 3), whereas the rest used alternative strategies to manage the symptoms (*i.e.*, Step 1 to Step 2).

#### Traditional herbal medicine

Six women, most of whom thought the symptom was a boil, visited traditional healers for herbal medicine, which they applied on the breast hoping the lump would disappear.

“When it [lump] initially started, I bought some herbal medication…and… smear it on the place… I thought it was a boil and when I take the drugs, it will eliminate it.” (P12)

#### Self-medication

Two women whose initial symptom was breast pain used over-the-counter medication to reduce the breast pain and stop the spread of the infection.

“It was Ampiclox and Paracetamol that were helping me. So whenever I took these, then the pains would reduce a little…and stop the infection.” (P22)

#### Faith healing

Two women who associated the symptoms to spiritual causes sought faith healing to manage the initial symptoms.

“A friend introduced me to a prayer group and so I joined…I put [prayer group anointed] oil on it.” (P32)

#### Wait and see

Nineteen women decided to monitor the symptoms since they were not disrupting their daily activities and they did not consider them serious enough to seek care.

“So when I observed the lump, I decided to wait awhile…because it wasn't causing me any pain. I could carry on my normal activities.” (P25)

### Step 3: Decision to seek orthodox medicine

Eventually all the women decided to seek orthodox medicine (*i.e.*, either Step 1 to Step 2 or Step 2 to Step 3) due to a combination of the following factors.

#### Additional symptoms

Ten women decided to seek allopathic medicine only when additional symptoms disrupted their daily activities. These additional symptoms included pain, lump enlargement, wound, increased breast swelling, and liquid oozing from the breast.

“The lump was tiny but later on, it was getting bigger and that was the reason that prompted me to come to [hospital].” (P27)

#### Alternative therapy failure

Some women decided to seek orthodox medicine when there was no improvement from the use of alternative therapy.

“Later, I realized the lump was getting bigger and when I touch the breast, it was more painful… I couldn't groom regularly…that was when I realized that I have to go to the hospital.” (P22)

#### Advice and pressure from social network

Other patients sought orthodox medicine due to advice from their social network. The advice could have been immediately after the patient saw the initial symptom, after failed alternative therapy or after they had additional symptoms.

“So when I told my husband, he said you are saying you have observed a lump…take it to the hospital.” (P18)

### Diagnostic interval

The average time between reaching the first health facility and getting a breast cancer diagnosis was 6 months (range 1–8 months).

### Step 4a: Reach first health facility

The health care system in Ghana includes both private and public hospitals. The public health care structure comprises three main levels: health centers and district hospitals that provide basic health care, regional hospitals that provide secondary health care, and teaching hospitals that provide tertiary care.^27^ The two major factors that influenced which initial health facility women sought care at were recommendation from social network and familiarity with the specific health care providers. Familiarity with specific health care providers could be due to information the women had heard from the media or where they sought regular medical care. Most women first sought medical care at health centers and district hospitals. Only eight women initially sought care at teaching hospitals. Three women initially sought care at one of the private hospitals. Once at the first health care facility, the women were examined and either (1) referred for diagnostic evaluations (*i.e.*, Step 4a to Step 5); or (2) referred to a higher-level facility that had breast cancer specialists (*i.e.*, Step 4a to Step 4b); or (3) misdiagnosed and went back home (*i.e.*, Step 4a to Step 1).

I felt the burning sensation and to [health center] who declared there was nothing wrong…I went back home. (P3)

### Step 4b: Reach second health facility

At the second health facility, women were either (1) referred for diagnostic evaluations (*i.e.*, Step 4b to Step 5), or (2) went back home due to misdiagnosis (*i.e.*, Step 4b to Step 1), or (3) referred to a third higher level facility for diagnosis (*i.e.*, Step 4b to Step 4c).

“[At district hospital], they made me take a scan… And then later on, I was told…they wouldn't be able to take care of me so I should come to [tertiary hospital].” (P12)

### Step 4c: Reach third health facility

One woman was referred to multiple facilities before she was diagnosed (*i.e.*, Step 4a to 4b to 4c to 5).

“I went to the nurses [in a health center] who advised going to see the doctor [at district hospital]…. He [doctor at district hospital] recommended being transferred because of how advanced my situation was. He directed me to [regional hospital]. However, we didn't go but came here instead [tertiary hospital].” (P4)

### Step 5: Diagnostic evaluations

None of the hospitals, including the tertiary hospitals, had all the necessary diagnostic services available. Patients had to be referred to private laboratories for diagnostic procedures such as mammogram. This process involved the women going back and forth from the referring hospital to the diagnostic laboratories for sample collection and back to the hospital to present the results.

“The staff of [tertiary hospital) required me to do a lot of labs… I was made to place my breast in a machine [mammogram] in [private lab] and I brought all the results back to them.” (P13)

### Step 6: Get diagnosed with breast cancer

Eventually all women were diagnosed with breast cancer. Twenty women were diagnosed at tertiary hospitals, three women were diagnosed at private hospitals, while the rest were diagnosed at regional and district hospitals. At the diagnosing facility, the women were examined and if the provider was suspicious of breast cancer, they would order diagnostic evaluations for her. These evaluations included mammogram, computed tomography (CT) scan, and biopsy.

“I did the mammogram and then the ultra sound… then biopsy. It was the biopsy that proved it that it was cancerous cells.” (P6)

Twenty-one women were missing stage information and 23 women were missing breast subtype information. Of the 10 women who had stage information, 60% had stage III disease, 20% had stage IV, and 20% had stage II. Of the eight women who had breast subtype information, 25% had human epidermal growth factor receptor 2-enriched subtype, 25% had luminal A, and 50% had triple-negative subtype. The women stated their initial reactions to the diagnosis included shock, fear of mastectomy and death, crying, and financial worry. However, others said they were not scared or worried as they leaned on their religious faith and encouragement from the doctor who delivered the diagnosis. Some patients continued to Step 7, whereas others went back to Step 1.

### Pretreatment interval

The average time between breast cancer diagnosis and starting treatment was 5 months (range 3 weeks–6 months).

### Step 7: Discussion at tumor board

KATH tumor board group consists of surgeons, pathologists, oncologists, radiologists, and nurses. After diagnosis, all patients are discussed by the tumor board to determine the treatment course. Patients are encouraged to be present when their cases are being discussed if at all possible.

“The doctor scheduled me for a meeting [tumor board]. The nurse brought me to this meeting. So after they [tumor board members] had asked me a few questions, they discussed it and started me on the chemo.” (P28)

### Step 8: Evaluations before starting treatment

After breast cancer diagnosis, the women typically needed more laboratory testing before they started treatment.

“I had to finish my labs; the full blood count, the kidney function test and everything before they started the treatment.” (P8)

### Treatment interval

#### Step 9: Start treatment

The most common treatment course was neoadjuvant chemotherapy, followed by surgery, followed by adjuvant chemotherapy and radiotherapy and hormone therapy.

“I was given six injections of chemotherapy… then they took me to cut off the breast [mastectomy]. After the operation I was put on a machine [radiotherapy], and after the machine, I was given a prescription for medication that I was buying [hormone therapy].” (P29)

## Discussion

This study describes Ghanaian women's paths from the moment they detect breast cancer-related symptoms through receipt of breast cancer treatment. To our knowledge, this is the first study to trace the breast cancer care pathway from Ghanaian women's perspectives. Similar to Kohler et al.^6^ study we found that the breast cancer pathway in Ghana is not linear. The Model of Pathways to Treatment framework,^9^ which we used to guide our data collection, is built on an assumption that women only use orthodox medicine to manage their breast cancer. However, in our study, we found that women frequently moved among different management approaches. We propose a modified framework (Fig. 1), which is grounded in findings from our interviews with Ghanaian women with breast cancer.

All the women discovered the symptoms themselves but only two women initially associated their symptoms with breast cancer. The rest thought their symptoms were not serious or could be easily managed by alternative therapies. This contributed to the substantially long average time of 11 months between detection symptoms and seeking orthodox medicine. The women in our study used different strategies to manage their breast cancer symptoms: traditional herbal medicine, over-the-counter medication, faith healing, and wait and see if the symptoms went away. Wait and see was the most common management strategy and involved the women monitoring their symptoms at home until they progressed to where the symptoms disrupted their ability to carry out daily activities. The main reasons for a wait and see approach were no pain associated with the symptom and interpretation that the symptoms were not serious and would resolve with time. This is consistent with previous research, which has shown that women who have symptoms that are not associated with pain and who interpret symptoms as not serious are less likely to seek care immediately.^18^

The second most common symptom management strategy was seeking care from traditional herbalists and faith healers (alternative therapy providers). In our study, the women who sought alternative therapies assumed their symptoms were due to common ailments such as a boil or were caused by evil powers. Alternative therapy providers are more accessible, thus, are more often consulted for common ailments. They are also more trusted, compared with orthodox medicine providers, to treat diseases whose cause is associated with spiritual powers.^28^ Alternative therapy is commonly used in Ghana and other sub-Saharan African countries for cancer management.^29^ However, the literature typically describes alternative therapy as a barrier to early detection and timely orthodox medicine receipt.^3,6^ To improve breast cancer management in this community, it is essential to acknowledge the critical role of alternative therapy in the breast cancer diagnosis and care pathways and develop approaches that integrate them into breast cancer diagnosis and management. In 2011, a plan for integrating alternative therapy and orthodox medicine for all diseases was launched in Ghana. However, there were no clear guidelines on what integration meant and how to go about it. For example, a study in one of the pilot hospitals for the integration found that patients who were seeking care at the hospital were not even aware that herbal services were also being provided. In addition, the orthodox medicine providers at the pilot hospital perceived both systems to be parallel instead of integrated.^30^

There is potential for successful integration of alternative therapy providers in breast cancer management in Ghana. Faith healers and traditional herbal medicine providers in Ghana have expressed interest in working collaboratively with orthodox medicine providers.^31,32^ Alternative therapy providers have previously been successfully integrated in HIV/AIDS and mental health care prevention and delivery.^33,34^ For the integration to be successful, there needs to be trust between orthodox and alternative therapy providers and an acknowledgment that both providers are complimentary.^35^ In an integrated system, alternative therapy providers can play two major roles: triage and offering psychosocial care and support. In the triage role, alternative therapy providers would immediately refer women with breast cancer-related symptoms to orthodox medicine. The psychosocial role of alternative therapy providers was also clear from our study, where some women consulted with faith healers for emotional support after the diagnosis.

All the women in our study detected the symptoms themselves. This is expected given Ghana does not have a national breast cancer screening program thus screening is *ad hoc*.^3^ However, there are nongovernmental organizations (NGOs) in Ghana that organize clinical breast examinations (CBE). CBE is the physical examination by a health care provider to check for breast abnormalities.^36^ CBE has been shown to lead to significant breast cancer downstaging.^37^ However, the CBE programs organized by the NGOs in Ghana are sporadic, which reduces their effectiveness. For example, one of the women in our study noticed the breast cancer-related symptoms in August. However, she was not aware of any hospitals that carried out breast cancer screening so she decided to wait for the annual October community breast cancer screening. For these programs to be effective, they need to be consistently available and women need to be aware of their locations.

Given the challenges of implementing national screening programs, a more cost-effective method to increase early diagnosis might be breast cancer awareness programs. Four women in our study sought medical care faster since they had learnt about breast cancer through the media and church events. In addition, a study in Ghana found that a community-based breast cancer awareness program significantly improved women's breast cancer knowledge and uptake of breast self-exam.^38^ Studies found that 60% of women in Ghana got health information from radio and television^39^ and that mass media awareness is a cost-effective intervention for increasing participation in breast cancer screening Ghana.^40^

Our study has several limitations. There is potential for selection bias. All our study participants were receiving breast cancer care, thus, we missed the experiences of women who never made it to the end of the breast cancer care pathway. Women who are not engaged in the cancer pathway may have had different experiences that we did not capture. However, we believe that the experiences of women who successfully navigated the various health care systems have insights that apply to those who were not successful. We asked women to remember all the steps that they took since symptom detection. Some of the women admitted they did not recall some of the events. Recall bias could also impact our study. For example, women may not have mentioned that they sought alternative therapies at some point in their pathway due to stigma. Strengths of our study include examining the pathway from the women's perspective and use of medical records to confirm procedures and treatment.

## Conclusions

Our study highlights the large number of steps that women go through from symptom detection to receiving orthodox breast cancer treatment in Ghana. Our framework can be used to identify locally relevant interventions that can be implemented to improve early detection and timely treatment of breast cancer in Ghana. Theoretical frameworks are important as they provide a systematic approach to understanding heath-seeking behavior by building on existing knowledge.^41–43^ However, few frameworks include the role of alternative therapy providers and are therefore not applicable to settings, where patients commonly seek care from these providers. We show that alternative therapy providers play a major role in the breast cancer care continuum in Ghana and alternative therapy and orthodox medicine are not mutually exclusive pathways that women take contrary to the way they are presented in the literature. We recommend integration of alternative therapy providers in breast cancer diagnosis and management strategies. Additional research is warranted to assess how incorporating alternative therapy providers improves early detection of breast cancer, access, and adherence to treatment. Lastly, findings from this study may be applicable to other countries where use of traditional medicine and faith healing is common.

## Supplementary Material

Supplemental data

## Abbreviations Used

CBE: clinical breast examinations
CT: computed tomography
HER2: human epidermal growth factor receptor 2
KATH: Komfo Anokye Teaching Hospital
NGOs: nongovernmental organizations
RA: Research Assistant
