# Supplementary material for: Pathways to Breast Cancer Diagnosis and Treatment Among Women in Ghana: A Qualitative Study
Source: Womens Health Rep (New Rochelle). 2021 Jul 16;2(1):234–44. doi: 10.1089/whr.2020.0117 (PMC8310750; doi:10.1089/whr.2020.0117)
Supplement: Supplemental data [file Supp_Data.docx]

**Supplementary Material**

**Interview Guide (Section 1)**

**Participant ID**

**Pathway to breast cancer care among women in Ghana patient interview questionnaire**

**Introduction:**

*Thank you for taking the time to talk to us about your journey seeking care for breast cancer. My name is (Research Assistant name) and this is Waruiru who is a student. Waruiru is working on research on the pathway to breast cancer care among women in Ghana. We hope that the information gathered from these interviews will help us better understand Ghanaian women’s journey in seeking care for breast cancer from symptom detection to treatment and ways to ensure that women get the best breast cancer care soonest possible.*

*We will be recording the session because we do not want to miss any of your input. We know you have helpful comments and we cannot write fast enough to get them all down. We will not include any names or other identifiable information and what you say is confidential. Do you have any questions for me? Do I have your permission to start the interview?*

**Icebreaker:**

What Ghanaian food do you enjoy making?

**Main question:**

**Please tell us the story of your journey from when you detected bodily change to when you started receiving breast cancer treatment**

**Below are the probes (only ask the probes if the patient did not provide this information)**

1. Please tell us the first change or symptom that you noticed in your breast?

- Time of discovery (date & year)
- Please tell us how you discovered the symptom or change in your breast?
- What happened after the discovery?
- What did you think the symptom was?
- Was it a serious issue to you?
- So what did you do after the discovery
  - Did you get any treatment related to the symptom?

1. Please tell us what you thought was the cause of the breast symptoms?

- Why did you think that was the cause?

1. Please tell us if you shared the information with anybody?

- Who?
- Why?
- When?
- What did the person tell you?

1. Did you see a doctor about the symptom?

- Where (facility)? Why this facility?
- When (date & year)?
- Why early? Or why delay?
- What happened at the hospital?
- What did the doctor say?
- What did the doctor do for you?
- Any labs?
- Any referral? Why, when and to where?

1. Please can you share with me the journey from when your symptom was assessed by the doctor to diagnosis?
   - What happened?
   - What were you asked to do?
   - The various investigations requested
   - Where did you do each of these?
   - cost
2. Were you actually told that you have breast cancer?

- When (Date)
- Who informed you? And Where?
- How did you feel
- So what happened next?
- Who did you inform about your breast cancer? Why?
- Any referral? By who? When and to where?

1. So have you received any treatment so far?
   - What type?
   - When did you start?
   - How long have you been on treatment?
2. Please can you share with me your experiences with the treatment so far and if there are factors that have impacted your ability to continue receiving treatment
   - Side effects
   - Cost
   - Any insurance
   - Any labs
   - How long it takes you to get to the facility
   - Any support?
3. *(Research Assistant summarize what the patient said to check accuracy)*
4. Is there any other thing you may want to share with us.

*Thank you for taking the time to share your journey with us. We now have a short survey that we would like you to fill. Please let us know if any question is not clear*

**Section 2**

Participant Study ID

| **Qn No.** | **Question** | **Answers** |
| --- | --- | --- |
| 1 | Please how old are you (years)? | Years: |
| 2 | What is your marital status | ☐ Single  ☐ Married/Partnered/Cohabiting  ☐ Widowed/Divorced/Separated |
| 3 | What is your highest level of education? | ☐ Primary school  ☐ High school  ☐ Technical college diploma  ☐ Bachelor degrees  ☐ Masters  ☐ Other (specify) |
| 4 | What is your religion | ☐ Christian  ☐ Muslim  ☐ Other (specify) |
| 5 | Are you employed? | ☐ Yes  ☐ No (SKIP to question 7) |
| 6 | If employed, who do you work for? | ☐ Government  ☐ Self-employed  ☐ Other (specify) |
| 7 | Do you have health insurance? | ☐ Yes  ☐ No |
| 8 | Has anyone in your family ever had cancer? | ☐ Yes  ☐ No (SKIP to question 11) |
| 9 | Your relation to them? | ☐ Father  ☐ Mother  ☐ Grandmother  ☐ Grandfather  ☐ Sibling  ☐ Child  ☐ Husband |
| 10 | If anyone in your family has had cancer, what type of cancer did they have? | ☐ Breast  ☐ Other |
